# Rheological Characteristics of Model Gluten-Free Dough with *Plantago* Seeds and Husk Incorporation

**DOI:** 10.3390/foods11040536

**Published:** 2022-02-13

**Authors:** Ewa Pejcz, Iva Burešová

**Affiliations:** 1 Department of Fermentation and Cereals Technology, Wrocław University of Environmental and Life Sciences, Norwida 25, 50-375 Wrocław, Poland; 2 Department of Food Technology, Tomas Bata University in Zlín, Vavrečkova 275, 760 01 Zlín, Czech Republic; buresova@utb.cz

**Keywords:** bread quality, physical properties, texture, shelf-life, *Plantago psyllium*, *Plantago ovata*

## Abstract

The seeds and husk of *Plantago* origin are rich source of dietary fiber known for its medicinal use. Despite the use of both *Plantago psyllium* and *Plantago ovata* products due to their physicochemical and nutritional properties, only the effects of *Plantago ovata* husk have been studied. Their structure-forming properties may positively affect gluten-free bread quality only if an adequate dough hydration is used. The aim of the work is to analyze the effect of different *Plantago* products: *Plantago psyllium* seeds and *Plantago ovata* seeds and husk in quantities of 3, 6 and 9% share on the rheological profile of model gluten-free dough and bread and bread’s technological quality and shelf-life. The rheological parameters of the dough were determined with Mixolab protocols and uniaxial deformation test. Bread quality and its textural profile analysis after cooling and storage were determined. The addition of *Plantago psyllium* seeds weakened the dough. All additives contributed to a reduction in starch retrogradation, bread hardness and water loss during baking, and to the improvement of the doughs’ resistance to extension, dough energy and bread yield. This influence is strongest when the *Plantago ovata* husk was used. However, the consumer acceptance of the tested breads was low and, in this respect, the breads with the addition of seeds of both *Plantago psyllium* and *ovata* were considered to be better than the husk.

## 1. Introduction

The genus *Plantago* includes over 200 species. The seeds known as psyllium are mainly sourced from black *Plantago psyllium* and blond *Plantago ovata* [1]. Plantago products are source of dietary fiber with a high proportion of water-soluble fraction having gel-forming properties [2,3,4]. Its effect on the human body includes cholesterol-lowering effect, laxative and glycemic index reduction [2,4,5,6,7]. This influence is due to its high-water absorption capacity and the ability to form viscous gels [2,8]. Psyllium mixed with water formulates a gel-like mucilage, the viscosity of which may affect the absorption of glucose, fat and cholesterol [9,10]. *Plantago* seeds are rich in mucilaginous substances composed mainly of arabinose, xylose, galacturonic acid and trace amounts of other sugars [1,2,5]. It is used as a food additive to upgrade the fiber content, texture, and rheological and sensory traits [11]. Despite the use of both *Plantago psyllium* and *Plantago ovata* products, only the effects of *Plantago* ovata husk, generally referred to as psyllium, have been widely studied.

In the case of bakery products, the effect of psyllium was investigated mainly in blends with wheat flour [3,12,13] and gluten-free blends [14,15,16,17,18]. The results of these studies indicate an increase in the dietary fiber concentration in the obtained products and the stability of psyllium at different pH levels and temperatures. The doughs with the addition of psyllium had a high water-binding capacity and improved workability [12,16,17], and affected starch gelatinization and retrogradation kinetics [3,14]. The physicochemical properties of psyllium may have a positive effect on the characteristics of bread made with its addition, such as volume, structure, appearance and shelf-life; however, this requires the use of an optimized degree of dough hydration [16,17,18,19].

To obtain the desired volume and texture of a bread, a strong network of dough components is required for gas retention, viscoelasticity and good dough rheology [20]. Gluten-free dough does not have the elastic and cohesive properties that non-gluten-free dough has, and it is more fluid than wheat dough, leading to poor dough formation with limited baking machinability. The bread made of it has a crumbling texture and tendency to rapid staling [21]. The model gluten-free dough and bread is based on rice flour due to its colorlessness, mild taste and low hypoallergenic properties [22]. Dough behavior during the bread making process is measurable by rheological methods that have been developed for wheat dough and adapted to be used for gluten-free dough. The results of rheological tests provide information about the dough functional behavior and structure and allow to determine the resulting bread quality [23]. Methods originally developed for wheat dough evaluation are also useful for gluten-free breads.

Due to the need to produce gluten-free bread for people with celiac disease, solutions are sought to improve its quality and consumer acceptability. A possible way to create a gluten-like physical dough structure is to enrich the dough with natural or synthetic dietary fiber containing complex carbohydrates [18]. Psyllium seeds and husk contain mostly arabinoxylans that make the material to which they are added highly water-absorbing and viscous [6]. The technological and nutritional functionality of the products derived from *Planatgo psyllium* and *Plantago ovata* is gaining interest. Plantago addition contributed to obtaining a softer crumb in high-fiber wheat bread [24] and improving dough mechanical properties and gluten-free bread shelf-life [25,26,27,28]. The aim of the study is to evaluate the performance of different *Plantago* products on the full profile of rheological properties of model rice-based gluten-free dough and bread as well as breads’ technological quality and shelf-life.

## 2. Materials and Methods

### 2.1. Materials

The study was performed using rice flour provided by Adveni Medical (Brno, Czech Republic). *Plantago psyllium* seeds, *Plantago ovata* seeds and *Plantago ovata* husk were purchased from a local herbal store and were milled in laboratory Hagberg Perten’s mill (Lab Mill type 120), yielding particles smaller than 300 µm. The basic composition of 100 g of raw materials declared by their producers is shown in Table 1. Sodium chloride, sucrose and freeze-dried yeast (Lesaffre Česko, Olomouc, Czech Republic) were also used for bread production. Rice flour and each of *Plantago* materials were blended at 3, 6 and 9% of rice flour substitution. Rice flour, without supplements, was used as the control sample.

### 2.2. Mixolab Tests

A Mixolab 2 (Chopin Technologies, Paris, France) device was used for the physical dough tests of rice flour and each blend of rice flour–*Plantago* materials at 3, 6 and 9%. Double measurements of the rheological behavior of flour during mixing and the double determination of the rheological behavior during mixing and heating were performed following 54-21.02 [29] and 54-60.01 [30] AACCI methods. The Mixolab analysis recorded dough resistance and temperature for every second of the measurement. The measurement results presented as a graph allow the calculation of the rheological parameters of the dough and are well described by Haratia et al. (2020) [31].

### 2.3. Uniaxial Deformation

A uniaxial deformation test was performed following the dough and gluten extensibility measurement method, using the Kieffer rig as described by Dunnewind et al. (2004) [32]. The doughs of flour blend and 2% salt addition were prepared using Mixolab at the time at the end of the dough development. Dough pieces were formed into 8 straps using a greased Teflon mold and left for 40 min at 24 ± 1 °C and 85% relative air humidity. The test was carried out using the TA.XT plus texture analyzer (Stable Micro System Ltd., Godalming, UK). The following test procedure for micro-extension trials on doughs using a 5 kg load cell was on mode measure force in tension with a return-to-start option, pre-test speed of 2.0 mm/s, test speed of 3.3 mm/s, post-test speed of 10.0 mm/s, distance of 75 mm and trigger force of 5 g. Based on the measurements, the following parameters were calculated: peak force [N], resistance to extension [N·mm], and distance at which this peak force occurs [mm]. The results are presented as mean values of eight replicates.

### 2.4. Bread Preparation and Evaluation

A dough was made from the flour blend, water, active dry yeast, salt and sucrose (Table 2). The amount of water was determined using Mixolab (Table 3). Dry yeast was reactivated for 6 ± 1 min in a sucrose solution prepared from 100 ± 1 mL of water (35 ± 1 °C) and 5.58 ± 0.02 g of sucrose. The flour blend (300 g), the solution, salt, and the rest of water were mixed in a Spar mixer (Spar Food Machinery MFG, Taiwan) for 6 min. After kneading, the dough was weighed (Ohaus PX224M, OHAUS Europe, Switzerland). Dough portions of 150 ± 5 g were scaled into bread pans and proofed for 20 ± 1 min at 30 ± 1 °C and 85% relative air humidity. The loaves were baked in an oven (MIWE cube, Pekass Plzen, Czech Republic) at 180 ± 5 °C for 20 ± 2 min, initially steamed for 10 s. The breads removed from the pans were cooled for 2 h at room temperature. Three batches of three samples were baked for each flour blend.

After cooling, the breads were weighed and the volume of breads was measured using plastic granulates of rape-seed size. Loaf volume was expressed in ml per 100 g of the flour blend used. Loaf specific volume (mL/g) was calculated by dividing the volume of the bread by its weight. The results are presented as mean values of three measurements of different loaves. Breads were stored for 24 h. Fresh and stored breads were subjected to consumer acceptance evaluation by ten panelists (male and female students of 19–24 years) using 9-point hedonic scale (where 1 meant “extremely dislike” and 9 meant “like very much”). The evaluated parameters were crust and crumb appearance and color, crumb softness, elasticity, porosity, and flavor. Overall acceptability was calculated as the average result of the assessed parameters of each of the objects.

Breads’ textural properties were evaluated in fresh breads (after 3 h of cooling) and after 24 h storage. Texture profile analysis (TPA) was performed using the TA.XT plus texture analyzer (Stable Micro System Ltd., Godalming, U.K.). Each of three samples of 35 mm diameter and 10 mm height cut from the center of each loaf was placed onto the analyzer and squeezed twice to 4 mm with cylinder probe P/75 of 1.00 mm/s speed. The crumb parameters were determined using Exponent Lite software and included hardness, stickiness, elasticity, cohesiveness and chewiness.

### 2.5. Statistical Analysis

The results were analyzed with Statistica 13.3 software package (StatSoft, Tulsa, OK, USA) with a one-way analysis of variance (ANOVA) test. The distribution of the data was normal as assessed by the chi-square test. Significant differences at *p* ≤ 0.05 between the mean values and homogeneous groups were determined using Duncan’s multiple range test. Significant Pearson’s correlation coefficients between the variables (significance level α ≤ 0.01) were determined (results in the supplementary data).

## 3. Results and Discussion

### 3.1. Mixolab Rheological Profiles

**Figure 1:**
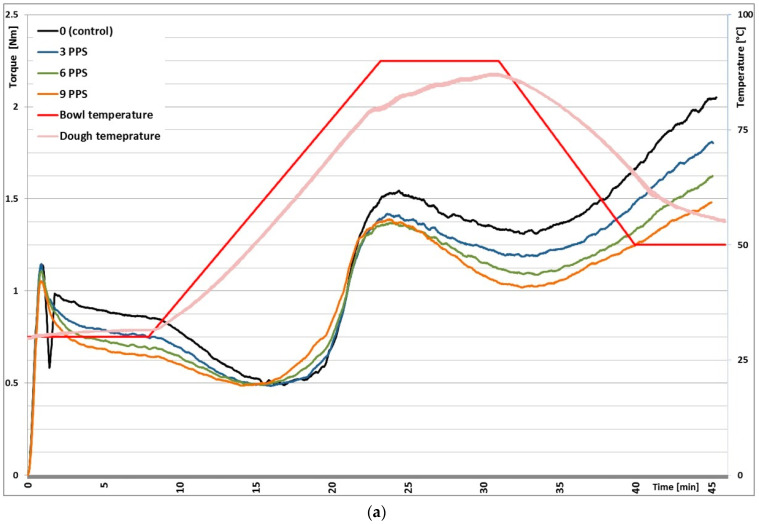

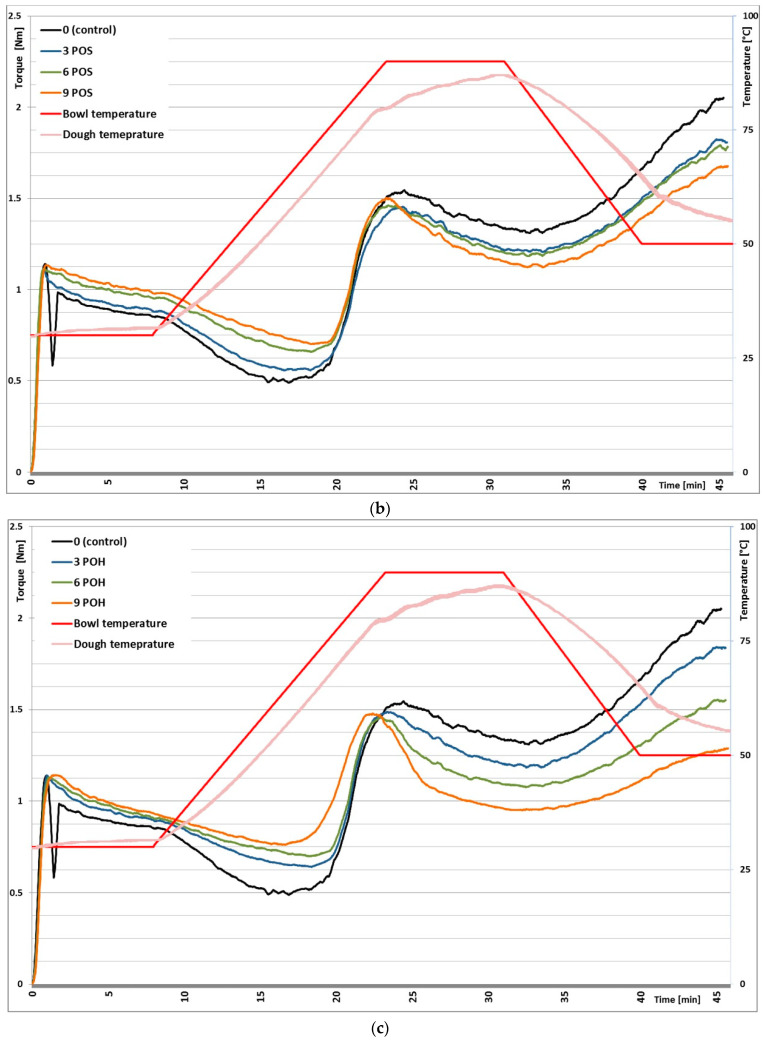
Mixolab profiles of rice flour without and with *Plantago* addition. (**a**) Effect of *Plantago psyllium* seeds (PPS). (**b**) Effect of *Plantago ovata* seeds (POS). (**c**) Effect of *Plantago ovata* husk (POH).

Table 3 shows the average results of farinographic tests conducted on rice flour blends with *Plantago psyllium* seeds (PPS), *Plantago ovata* seeds (POS) and *Plantago ovata* husk (POH) at 0, 3, 6 and 9% levels of substitution. Rice flour dough had a hydration of 61.2%, a dough development time of 2.3 min, a dough stability of 1.45 min, a dough softening of 95 FU and a mixing tolerance index (MTI) of 128.4 FU. Both during the analysis of the farinographic profile and the full Mixolab profile, the resistance of the dough made of rice flour alone decreased in the first minutes of mixing. The resistance then stabilized after the rice proteins were hydrated reaching the target dough torque. This effect was eliminated with the use of *Plantago* additives, which allowed the water to be evenly distributed in the dough in the first stage of mixing (Figure 1). Mixolab water absorption indicates the amount of water needed for obtaining the dough of the consistency of 1.1 ± 0.05 N·m. Each of the additives significantly increased the water absorption of the blend compared to the control, which was rice flour without any addition. Blends with POH incorporation had the highest water absorption, while the lowest were those with PPS. At 9% of the rice flour substitution, the hydration increased by 22.4% (PPS), 23.9% (POS) and 60.3% (POH). According to Santos et al. (2020) [26], the dough resistance at each stage of the Mixolab assay increases with the addition of psyllium if the dough hydration is not increased sufficiently. Thanks to psyllium’s ability to gel and absorb water an increased farinographic water absorption along with an increased proportion of Psyllium have also been observed in studies conducted by Kamaljit et al. (2011) [12] and Mariotti et al. (2009) [17]. Ferrero (2017) [33] showed that the type of hydrocolloid used had a greater impact on water absorption than its share in the dough. Other farinographic parameters, such as dough development time and dough stability, indicate the flour’s strength. The addition of PPS did not affect dough development time. POS and POH additives extended dough development time along with their increasing share. With 9% POS addition, the dough development time was extended from 2.3 to 5.1 min and with POH to 8.8 min. The increase in dough development time was caused by an increased amount of fiber content, which requires longer water absorption [21]. The dough stability was extended only in the case of 9% POH rice flour substitution (from 1.45 to 2.50 min); in other blends, the stability of the dough did not change when compared with the control. The increasing share of PPS contributed to the increased dough softening (to 6% addition) and increasing mixing tolerance index (up to 9% addition). Mixing tolerance index (MTI) is the difference in FU between the top of the curve at the peak and the curve position measured 5 min after reaching the peak; therefore, higher MTI values mean lower resistance of the dough towards mechanical damage [13]. Sim et al. (2015) [13] observed a significant increase in MTI with the addition of non-starch polysaccharides to the dough. On the other hand, in the present study, the increasing share of POS and POH in flour blends resulted in a decrease in both dough softening and MTI. This means that the gels formed by both the seed and the husk of *Plantago ovata* are strong and resistant to mechanical deformation.

The results of the remaining parameters based on the complete Mixolab profile are presented in Table 4. Graphs made with Mixolab are shown in Figure 1. Point C2 measures protein weakening due to mechanical work and increasing temperature. In this study, PPS incorporation, regardless its level, resulted in lowering the point C2. This confirms that doughs with this additive was less resistant to mixing and temperature. On the other hand, with the increasing share of POS (up to 6%) and POH (up to 9%), C2 points were higher, confirming the stability of their mucilage. Cappa et al. (2013) [15] and Mariotti et al. (2009) [17] observed improved dough workability with psyllium addition to the gluten-free blends as the network structure of the added hydrocolloids affects dough rheological behavior. Mariotti et al. (2009) [17] observed an improvement in the physical properties of the dough with the addition of psyllium resulting from the formation of a film-like structure and a continuous protein phase, as visualized by scanning electron microscopy and confocal laser scanning microscopy. Current research indicates that the *Plantago ovata* seeds strengthened the structure of the dough similar to the husk. *Plantago psyllium* seeds, on the other hand, caused the opposite effect. Point C3 measures starch gelatinization, point C4 indicates hot gel stability and point C5 retrogradation of starch in the cooling phase. The dough pasting properties and potential staling trends of bread are shown in Table 4. Dough pasting properties (especially peak viscosity at C3 and C5–C4 setback) correlate with bread staling kinetics [34].

Each of the additives used contributed to the reduction in the dough resistance at point C3, at its greatest extent after the addition of PPS. A decrease in the value of the C4 point was also observed along with an increasing share of all additives, the strongest in the case of PPS and POH. The value of C3 depends on the starch characteristics and amylase activity of the sample; decreased resistance of the dough at the C3 and C4 point means the increase in the activity of amylolytic enzymes. A significant reduction in the point C5 level was also shown along with an increase in the share of each of the additives. The highest decrease was recorded for POH (from 2.008 to 1.277 N·m), PPS to 1.376 N·m, and the lowest for POS to 1.568 N·m. The use of materials from the *Plantago* genus contributed to reducing the tendency of the dough to retrograde (C5–C4). The reduced rate of starch retrogradation was particularly evident with the increasing proportion of POH in the blends. The staling tendency of bread during storage is strongly correlated with starch gelatinization properties, especially with peak viscosity and setback [34]. Cappa et al. (2013) [15] and Mancebo et al. (2015) [16] showed that, if the dough is well hydrated, the soluble fiber from psyllium can soften the crumb, while insufficient hydration results in crumb hardening. The results of Aprodu and Banu (2015) [14] showed that, when the amount of water was insufficient, the addition of psyllium increased the C4 and C5 parameters, while with the increased amount of water, they were lower. They explain this by the competition for water by starch and fiber. The addition of psyllium to the gluten-free dough results in the formation of a thin protein–hydrocolloid network, which contributes to the reduction in starch swelling and gelatinization. High water-binding capacity, the improvement of its distribution and retention contribute to the delay of starch retrogradation, improving the shelf-life of bread [13,15,17].

### 3.2. Uniaxial Deformation

The addition of all *Plantago* materials affected the behavior of rice flour dough during uniaxial deformation (Table 5). The rice flour dough, which was the control sample, had a resistance to extension of 0.123 N, dough energy of 0.863 N·mm and extensibility of 10.806 mm. These values are very low compared to those of the wheat flour for which this determination is usually performed, proving the low elasticity of the dough from rice flour alone. A dough suitable for bread making must have a sufficient strength to be able to stretch during expansion due to fermentation gases. The peak force, i.e., resistance to extension, increased under the influence of increasing proportions of *Plantago* additives. Their 9% shares improved dough resistance to extension at 87% (PPS), 158% (POS) and 290% (POH). The energy of the dough also increased under the influence of the increasing addition of products derived from *Plantago ovata*. The 9% of seeds (POS) incorporation increased the energy of the dough by 128% and the husks (POH) by 237%. The addition of *Plantago psyllium* seeds (PPS) had no effect on the dough energy. None of the *Plantago* additives used had a significant effect on the dough extensibility. The rice flour dough was unable to stretch sufficiently, which is needed for leavened baked products. A strong network of the dough components is required for the gas retention. The increase in the resistance to extension and dough energy indicate an improvement in dough behavior, especially under the influence of *Plantago ovata* products incorporation and the effect was dependent on the additive concentration. Improving the elastic properties of the dough allows the volume of the dough to increase during fermentation due to gas entrapment [35].

### 3.3. Bread Quality

**Figure 2:**
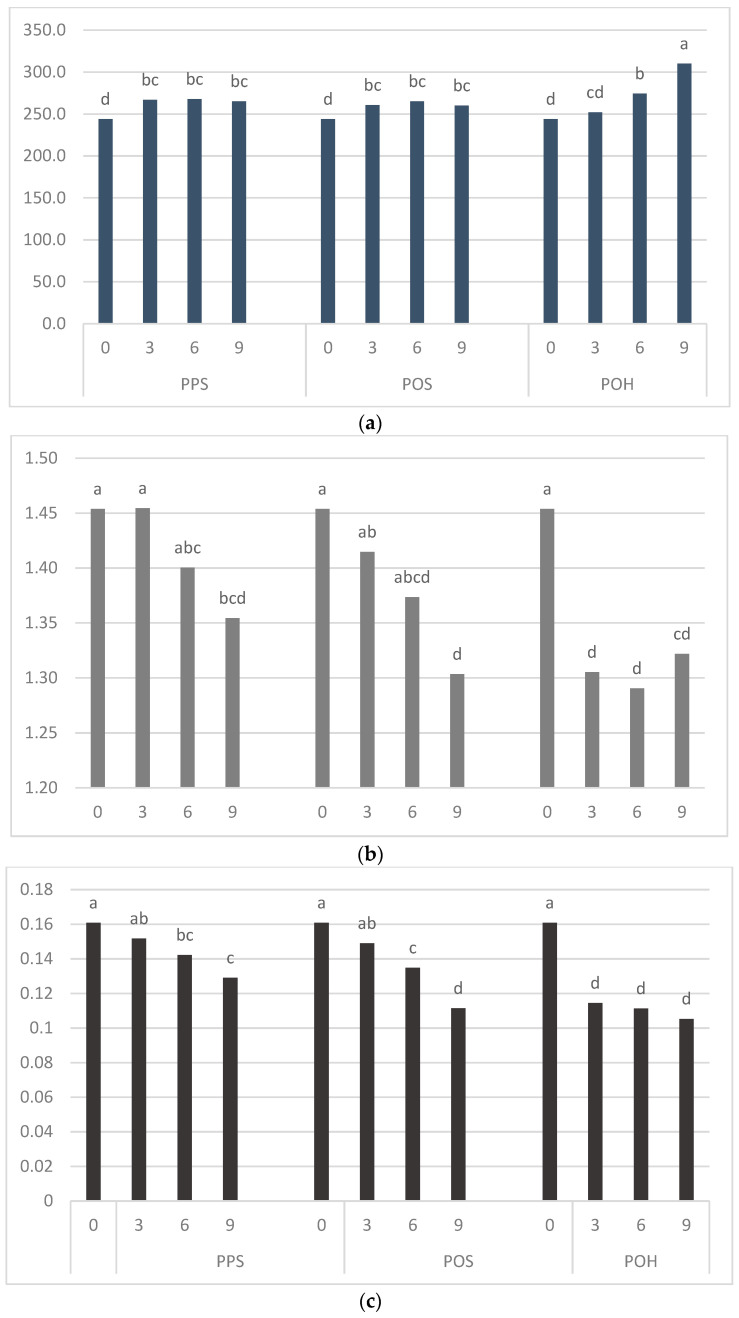
The quality parameters of rice flour–Plantago breads. Values represent the means of three replicates. Small letters (a, b, c, etc.) in the same column denote significant differences according to Duncan’s test (*p* ≤ 0.05). PPS: *Plantago psyllium* seeds; POS: *Plantago ovata* seeds; POH: *Plantago ovata* husk. (**a**) Loaf volume per 100 g of flour blend (cm^3^). (**b**) Specific volume (mL/g). (**c**) Water loss (%).

The quality parameters of gluten-free bread formulations containing different PPS, POS and POH levels are shown in Figure 2. *Plantago* incorporation had a strong influence on bread quality. The volume of bread obtained from 100 g of flour or a flour blend determines its technological efficiency. This volume was increased by all the applied *Plantago* additives. The POH addition affected the bread quality to the greatest extent. The use of both *Plantago psyllium* and *Plantago ovata* seeds (PPS and POS) resulted in an increase in the volume of bread obtained from 100 g of flour to a small extent and regardless of their share in the blends. On the other hand, the increasing addition of *Plantago ovata* husk (POH) resulted in a gradual increase in this parameter up to 27% with a 9% share of POH. The bread volume indicates how thin the dough structure may be stretched [13,16]. Aprodu and Banu (2015) [14] also observed an increase in the loaf volume as a result of adding psyllium. The gelling ability of psyllium hydrocolloids allows the structure of the dough with its addition to strengthen the gas cells and support their expansion, leading to an increase in the bread volume [33]. Loaf specific volume (ml/g) indicate the ratio of the bread’s volume to its weight. Due to the use of bread recipes that take into account the water absorption of the blends, higher water retention of blends made the breads heavier. Therefore, the use of *Plantago* products contributed to a reduction in the bread’s loaf specific volume. As in the case of blends’ water absorption, the greatest effect was observed for POH and the lowest for PPS. In the results of Fratelli et al. (2018) [10], it was possible to improve the loaf specific volume of gluten-free bread with psyllium addition using optimized dough hydration. Kamaljit et al. (2011) [12], Mancebo et al. (2015) [16] and Sim et al. (2015) [13] also described the decrease in the specific volume of bread when POH was added to the dough. Water loss during baking (%) decreased with the use of *Plantago* additives, proving their strong water-holding capacity. The increasing PPS and POS additives resulted in a gradual decrease in water loss, while the addition of POH significantly reduced water loss, regardless of the amount used.

**Figure 3:**
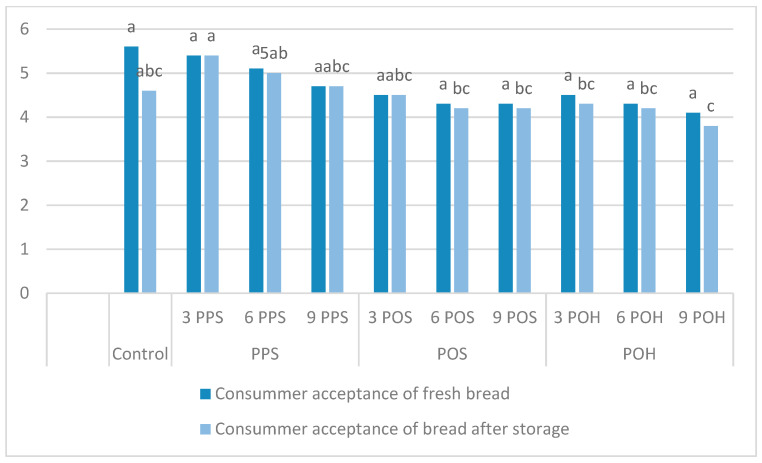
The consumer acceptance of fresh and stored rice flour–Plantago breads. Values represent the means of ten replicates. Small letters (a, b, c, etc.) in the same column denote significant differences according to Duncan’s test (*p* ≤ 0.05). PPS: *Plantago psyllium* seeds; POS: *Plantago ovata* seeds; POH: *Plantago ovata* husk.

The consumer acceptance of fresh bread and after storage expressed on a 9-point hedonic scale is presented in Figure 3. Despite the lack of statistically significant differences between the scores of fresh bread, it can be noticed that all of the used *Plantago* additives resulted in deterioration of product acceptance compared to the control rice bread. Among the *Plantago*-enriched bread, the highest rating was given to bread with 3% PPS and the lowest with 9% POH. Gupta et al. (2014) [3] also reported a decrease in overall quality score with addition of 5 g/100 g of POH. Using the *Plantago ovata* husk incorporation to gluten-free bread of a level up to 3 g/100 g by Zandonadi et al. (2009) [18] resulted in a good acceptance by individuals with and without celiac disease, and Kamaljit et al. (2011) [12] observed a better overall acceptability of breads with 3% POH incorporation than control. After 24 h of storage the breads with 3 and 6% of PPS incorporation received a higher score than the control bread, while lower scores were given to samples with POH and 9% POS. Comparing the ratings of fresh and stored bread, it was observed that the addition of seeds (PPS and POS) did not contribute to the deterioration of the acceptance of the bread after storage, which occurred in the case of the control rice bread and with the addition of husk (POH).

### 3.4. Texture Profile Analysis

The textural properties of fresh and stored breads are shown in Table 6. The hardness of the fresh bread with all the used *Plantago* additives was lower than that of the bread without their incorporation. The elastic properties of *Plantago*-enriched dough allow the dough to entrap gases, decreasing the breads’ hardness. The increasing share of PPS successively decreased the hardness of the fresh bread. After storage, the breads with the addition of PPS had a lower hardness than the control bread, but it increased with the increasing share of PPS. Bread made from rice flour alone and with a 3% PPS content decreased its hardness after storage and with 6 and 9% PPS increased its hardness within 24 h of storage. The POS incorporation to bread caused a decrease in fresh and stored breads’ hardness compared to control; however it was increasing together with the increasing share of POS. The addition 6 and 9% of POH significantly decreased the hardness of fresh and stored breads. According to research by Cappa et al. (2013) [15], Mariotti et al. (2009) [17] and Santos et al. (2020) [26], a higher water content in the dough, such as in the case of dough with 6 and 9% POH, may help to keep the bread crumb soft during storage. The lower hardness of bread crumb may be also related to lower setback values [16]. Breads’ cohesiveness is the ability to withstand compressive or tensile stress. It was not strongly affected by *Plantago* seeds (PPS an POS) incorporation, a significant increase in breads’ cohesiveness was observed only with the 9% addition of POH. A slight reduction in cohesiveness after storage was observed for control bread, and with 9% of PPS and 3% of POH incorporation. Springiness is the ability of the crumb of the bread to spring back after deformation during the first compression. Fresh breads’ springiness increased slightly with the use of *Plantago* additives (with the exception of 3% POS); after storage, breads with 6 and 9% POH addition were characterized by higher springiness than the others. Breads’ chewiness was decreased by *Plantago* products incorporation, mostly with the use of 3% of POS and 9% of POH. In the case of the control bread and the those with the addition of 3 and 6% of PPS and 3% of POH, a reduction in the chewiness of the breads after storage was observed. In the remaining samples (9% PPS, each with POS addition and 6 and 9% POH), the chewiness after storage increased. The results of Filipčev et al. (2021) [25] indicate that the addition of psyllium caused a reduction in the crumb hardening rate of buckwheat–carob bread and Santos et al. (2021) [27] observed that psyllium addition to gluten-free bread delayed the loss of its cohesiveness and springiness. The *Plantago* additives used did not significantly affect the resilience of breads’ crumb.

Significant Pearson’s correlation coefficients between the variables are shown in Table S1 of the supplementary data. A significant, positive correlation was found between farinographic parameters, water absorption, dough development time and dough stability, as well as between dough softening and MTI. Dough hydration was positively correlated with the C3–C4 setback, dough resistance to extension, dough energy and bread volume per 100 g of flour and negatively with Mixolab torque at C4, C5, C5–C4 setback, water loss during baking and hardness of fresh bread. This confirms the positive effect of using highly water-absorbing additives for gluten-free bread on the improvement of dough elasticity and reduction in bread’s hardness. Dough resistance to extension was positively correlated with the C3–C4 setback denoting the rate of amylolysis. There was also a positive correlation between the value of the dough resistance at point C2 (protein weakening during mixing and heating) and the dough energy. Bread hardness was related to the value of the dough resistance at the C5 point, which was responsible for the retrogradation of starch during cooling.

## 4. Conclusions

The use of seeds and husk from *Plantago ovata* strengthened the structure of the dough as measured by farinograph. The addition of *Plantago psyllium* seeds, in addition to the water absorption capacity, weakened the dough. Both *Plantago psyllium* and *Plantago ovata* (seeds and husk) additives contributed to a reduction in the starch retrogradation, bread hardness and water loss during baking, and to improve the doughs’ resistance to extension, its energy and bread yield. This influence is strongest when the *Plantago ovata* husk was used. The incorporation of *Plantago* products in gluten-free bread may be beneficial because of its potential to produce functional foods. However, due to the low consumer acceptance of the breads (especially when using high concentrations and POH), further research may focus on optimizing the recipes of the breads in order to improve their quality.

## Figures and Tables

**Table 1:** The composition of 100 g of raw materials.

	Rice Flour	*Plantago psyllium* Seeds	*Plantago ovata* Seeds	*Plantago ovata* Husk
Energy value	1484 kJ/349 kcal	1133 kJ/275 kcal	1133 kJ/275 kcal	749 kJ/178 kcal
Fat	0.6 g	7.2 g	7.3 g	0.0 g
Saccharides	79.0 g	63.1 g	64.0	77.4 g
Protein	7.0 g	14.4 g	14.0 g	0.4 g

**Table 2:** Gluten-free brad formulation per 100 g of flour blend.

Sample	Rice Flour	*Plantago* Addition	Sucrose	Yeast	Salt
Control	100	0	1.86	1.80	1.5
3 PPS	97	3	1.86	1.80	1.5
6 PPS	94	6	1.86	1.80	1.5
9 PPS	91	9	1.86	1.80	1.5
3 POS	97	3	1.86	1.80	1.5
6 POS	94	6	1.86	1.80	1.5
9 POS	91	9	1.86	1.80	1.5
3 POH	97	3	1.86	1.80	1.5
6 POH	94	6	1.86	1.80	1.5
9 POH	91	9	1.86	1.80	1.5

PPS: Plantago psyllium seeds; POS: Plantago ovata seeds; POH: Plantago ovata husk.

**Table 3:** Farinographic characteristics of rice flour–*Plantago* products blends.

Blend		Hydration (%)	Dough Development Time (min)	Dough Stability (min)	Dough Softening (FU)	Mixing Tolerance Index (FU)
**Control**		61.2 ± 0.49 g	2.30 ± 0.14 g	1.45 ± 0.07 b	95.0 ± 2.66 c	128.4 ± 0.74 d
PPS	3%	70.3 ± 1.34 f	2.45 ± 0.07 fg	1.45 ± 0.07 b	103.5 ± 0.71 b	156.5 ± 1.88 c
	6%	73.7 ± 1.44 d	2.55 ± 0.07 fg	1.45 ± 0.07 b	116.0 ± 1.41 a	164.4 ± 2.13 b
	9%	74.9 ± 1.46 cd	2.55 ± 0.21 fg	1.45 ± 0.07 b	116.5 ± 2.12 a	174.2 ± 0.63 a
POS	3%	70.8 ± 0.71 ef	3.55 ± 0.07 e	1.55 ± 0.07 b	88.0 ± 1.41 de	109.4 ± 0.52 f
	6%	74.6 ± 0.64 cd	4.25 ± 0.07 d	1.55 ± 0.07 b	87.0 ± 1.41 e	97.9 ± 1.13 g
	9%	75.8 ± 0.62 c	5.10 ± 0.14 c	1.60 ± 0.14 b	87.0 ± 1.41 e	91.0 ± 0.90 h
POH	3%	71.9 ± 0.85 de	2.65 ± 0.07 f	1.55 ± 0.07 b	94.5 ± 2.12 cd	120.1 ± 0.26 e
	6%	84.5 ± 1.41 b	6.55 ± 0.07 b	1.65 ± 0.21 b	93.5 ± 1.41 cde	109.1 ± 0.17 f
	9%	98.1 ± 1.89 a	8.80 ± 0.14 a	2.50 ± 0.28 a	88.5 ± 0.71 cde	98.6 ± 0.63 g

Values represent the means of two replicates. Small letters (a, b, c, etc.) in the same column denote significant differences according to Duncan’s test (*p* ≤ 0.05). PPS: *Plantago psyllium* seeds; POS: *Plantago ovata* seeds; POH: *Plantago ovata* husk.

**Table 4:** Mixolab profiles of rice flour–*Plantago* products blends.

Blend		C2 (N·m)	C3 (N·m)	C4 (N·m)	C5 (N·m)	C5–C4 (N·m)
**Control**		0.578 ± 0.007 d	1.613 ± 0.028 a	1.367 ± 0.019 a	2.008 ± 0.006 a	0.642 ± 0.013 a
PPS	3%	0.482 ± 0.003 e	1.390 ± 0.042 d	1.204 ± 0.025 b	1.678 ± 0.016 c	0.475 ± 0.040 b
	6%	0.485 ± 0.006 e	1.360 ± 0.016 d	1.090 ± 0.003 de	1.518 ± 0.008 e	0.428 ± 0.011 bc
	9%	0.483 ± 0.004 e	1.396 ± 0.010 d	1.020 ± 0.003 e	1.376 ± 0.002 g	0.356 ± 0.003 cd
POS	3%	0.583 ± 0.036 d	1.447 ± 0.010 c	1.220 ± 0.020 b	1.723 ± 0.004 b	0.503 ± 0.016 b
	6%	0.655 ± 0.006 c	1.472 ± 0.014 bc	1.188 ± 0.007 bc	1.674 ± 0.010 c	0.486 ± 0.017 b
	9%	0.700 ± 0.002 b	1.514 ± 0.015 b	1.110 ± 0.017 cd	1.568 ± 0.013 d	0.458 ± 0.004 b
POH	3%	0.655 ± 0.021 c	1.484 ± 0.006 bc	1.116 ± 0.025 cd	1.716 ± 0.008 b	0.601 ± 0.044 a
	6%	0.693 ± 0.008 b	1.479 ± 0.030 bc	1.094 ± 0.025 de	1.455 ± 0.011 f	0.361 ± 0.013 cd
	9%	0.772 ± 0.017 a	1.483 ± 0.004 bc	0.977 ± 0.039 f	1.277 ± 0.016 h	0.300 ± 0.040 d

Values represent the means of two replicates. Small letters (a, b, c, etc.) in the same column denote significant differences according to Duncan’s test (*p* ≤ 0.05). PPS: *Plantago psyllium* seeds; POS: *Plantago ovata* seeds; POH: *Plantago ovata* husk.

**Table 5:** The behavior of rice flour–*Plantago* products dough under uniaxial deformation.

Blend		Resistance to Extension (N)	Dough Energy (N·mm)	Dough Extensibility (mm)
**Control**		0.123 ± 0.007 f	0.863 ± 0.016 d	10.806 ± 1.291 a
PPS	3%	0.136 ± 0.016 ef	0.885 ± 0.014 d	9.099 ± 1.268 a
	6%	0.183 ± 0.018 cde	0.869 ± 0.013 d	10.664 ± 0.479 a
	9%	0.230 ± 0.026 c	0.976 ± 0.021 d	13.930 ± 1.274 a
POS	3%	0.165 ± 0.016 def	1.140 ± 0.018 d	11.543 ± 1.152 a
	6%	0.224 ± 0.012 c	1.539 ± 0.025 c	11.795 ± 1.284 a
	9%	0.318 ± 0.036 b	1.964 ± 0.022 b	11.130 ± 0.726 a
POH	3%	0.214 ± 0.014 cd	1.452 ± 0.016 c	12.273 ± 1.488 a
	6%	0.342 ± 0.016 b	2.049 ± 0.028 b	11.617 ± 1.097 a
	9%	0.480 ± 0.026 a	2.905 ± 0.026 a	12.020 ± 1.489 a

Values represent the means of eight replicates. Small letters (a, b, c, etc.) in the same column denote significant differences according to Duncan’s test (*p* ≤ 0.05). PPS: *Plantago psyllium* seeds; POS: *Plantago ovata* seeds; POH: *Plantago ovata* husk.

**Table 6:** The texture parameters of fresh and 24 h stored rice flour–*Plantago* breads.

Blend		Hardness (N)		Cohesiveness (%)		Springiness (%)		Chewiness (-)		Resilience (%)	
		Fresh	Stored	Fresh	Stored	Fresh	Stored	Fresh	Stored	Fresh	Stored
**Control**		26.73 ± 2.21 a	23.85 ± 2.30 ab	0.73 ± 0.04 b	0.68 ± 0.04 bc	0.69 ± 0.03 b	0.68 ± 0.04 b	13.45 ± 1.12 a	10.94 ± 0.79 abcd	0.44 ± 0.03 a	0.39 ± 0.04 ab
PPS	3%	22.00 ± 2.78 ab	14.65 ± 1.37 d	0.72 ± 0.02 b	0.72 ± 0.08 abc	0.73 ± 0.03 ab	0.76 ± 0.05 b	11.34 ± 1.09 ab	8.04 ± 0.43 d	0.40 ± 0.01 a	0.41 ± 0.06 ab
	6%	16.95 ± 2.58 bcd	17.88 ± 0.15 cd	0.76 ± 0.02 ab	0.74 ± 0.04 ab	0.80 ± 0.07 ab	0.74 ± 0.09 b	10.46 ± 1.55 abc	9.99 ± 1.04 abcd	0.45 ± 0.02 a	0.44 ± 0.02 a
	9%	13.89 ± 1.18 cd	20.51 ± 1.28 bc	0.75 ± 0.03 ab	0.68 ± 0.02 bc	0.80 ± 0.08 ab	0.72 ± 0.05 b	8.43 ± 1.05 bcd	13.41 ± 1.61 a	0.44 ± 0.01 a	0.37 ± 0.03 b
POS	3%	11.62 ± 1.65 de	17.48 ± 1.75 cd	0.75 ± 0.05 ab	0.73 ± 0.04 abc	0.70 ± 0.09 b	0.66 ± 0.08 b	6.14 ± 1.10 cd	8.41 ± 0.83 cd	0.47 ± 0.05 a	0.43 ± 0.03 a
	6%	14.31 ± 1.86 cd	17.59 ± 1.39 cd	0.77 ± 0.06 ab	0.73 ± 0.03 abc	0.72 ± 0.05 ab	0.73 ± 0.07 b	7.97 ± 0.84 cd	9.34 ± 0.79 bcd	0.46 ± 0.04 a	0.42 ± 0.01 ab
	9%	19.81 ± 0.16 bc	23.44 ± 1.72 ab	0.79 ± 0.02 ab	0.72 ± 0.00 abc	0.73 ± 0.04 ab	0.78 ± 0.05 b	11.46 ± 1.04 ab	13.18 ± 0.66 a	0.45 ± 0.03 a	0.40 ± 0.01 ab
POH	3%	21.87 ± 2.71 ab	24.75 ± 1.90 a	0.79 ± 0.03 ab	0.66 ± 0.02 c	0.77 ± 0.08 ab	0.77 ± 0.04 b	13.09 ± 1.25 ab	12.68 ± 1.38 ab	0.47 ± 0.02 a	0.37 ± 0.04 ab
	6%	15.26 ± 2.20 bcd	19.26 ± 1.44 c	0.79 ± 0.02 ab	0.75 ± 0.03 ab	0.82 ± 0.10 ab	0.82 ± 0.09 ab	9.93 ± 0.65 abc	11.74 ± 1.45 abc	0.45 ± 0.01 a	0.42 ± 0.01 ab
	9%	5.89 ± 0.39 e	9.00 ± 0.26 e	0.81 ± 0.01 a	0.77 ± 0.02 a	0.88 ± 0.11 a	0.86 ± 0.09 ab	4.28 ± 0.81 d	5.98 ± 0.43 e	0.43 ± 0.04 a	0.40 ± 0.03 ab

Values represent the means of three replicates. Small letters (a, b, c, etc.) in the same column denote significant differences according to Duncan’s test (*p* ≤ 0.05). PPS: *Plantago psyllium* seeds; POS: *Plantago ovata* seeds; POH: *Plantago ovata* husk.

## Data Availability

Results are available from the corresponding author.

## Supplementary Materials

The following supporting information can be downloaded at: https://www.mdpi.com/article/10.3390/foods11040536/s1. Table S1: Significant Pearson’s correlation coefficients (significance level α ≤ 0.01).
