# Supplementary material for: Rheological Characteristics of Model Gluten-Free Dough with Plantago Seeds and Husk Incorporation
Source: Foods. 2022 Feb 13;11(4):536. doi: 10.3390/foods11040536 (PMC8870862; doi:10.3390/foods11040536)
Supplement: Supplementary file 1 [file foods-11-00536-s001.zip › foods-1590907-supplementary.pdf]

Table S1. Significant Pearson's correlation coefficients (significance level $\alpha \leq 0.01$)

	H	DDT	DSt	DSo	MTI	C2	C3	C4	C5	C3-C4	C5-C4	RE	DEn	DEx	LV	SV	WL	FBH	FBC	FBS	FBCh	FBR	SBH	SBC	SBS	SBCh	SBR
**H**		0.910	0.843					-0.78	-0.846	0.770	-0.787	0.847	0.735		0.917		-0.760	-0.750									
**DDT**			0.857			0.829				0.716		0.862	0.794		0.806												
**DSt**						0.711				0.726		0.758			0.801												
**Dso**					0.949	-0.788																					
**MTI**						-0.890							-0.702														
**C2**												0.778	0.820				-0.701										
**C3**																											
**C4**									0.929	-0.776	0.715						0.777										
**C5**											0.923				-0.777			0.737									
**C3-C4**												0.803				-0.708	-0.880										
**C5-C4**															-0.765												
**RE**													0.943				-0.819										
**Den**																	-0.752										
**Dex**																											
**LV**																							-0.704				
**SV**																	0.863										
**WL**																											
**FBH**																					0.936						
**FBC**																											
**FBS**																											
**FBCh**																											
**FBR**																											
**SBH**																										0.877	
**SBC**																											0.716
**SBS**																											
**SBCh**																											
**SBR**																											

H- hydration, DDT- dough development time, DSt- dough stability, DSo- dough softening, MTI- mixing tolerance index, C2-C5- Mixolab torqe at points C2-C5, RE- dough resistance to extension, Den- dough energy, DEx- dough extensibility, LV- loaf volume 100g of flour blend, SV- specific volume, WL- water loss, FBH- fresh bread hardness, FBC- fresh bread cohesiveness, FBS- fresh bread springiness, FBCh- fresh bread chewiness, FBR- fresh bread resilience, SBH- stored bread hardness, SBC- stored bread cohesiveness, SBS- stored bread springiness, SBCh- stored bread Chewiness, SBR- stored bread resilience
